# An innovative thread-lift technique for facial rejuvenation and complication management: A case report

**DOI:** 10.1097/MD.0000000000010547

**Published:** 2018-05-25

**Authors:** Songjia Tang, Zhongxin Sun, Xiaoxin Wu, Yu-Yan Wang, Jufang Zhang

**Affiliations:** a Plastic and Aesthetic Surgery Department of Hangzhou First People's Hospital; b State Key Laboratory for Diagnosis and Treatment of Infectious Diseases, Collaborative Innovation Center for Diagnosis and Treatment of Infectious Diseases, The First Affiliated Hospital, School of Medicine, Zhejiang University, Hangzhou, China.

**Keywords:** facial aging, facial rejuvenation, small incision rhytidectomy, small needle knife, thread lift

## Abstract

**Rationale:**

Aging of face is an unavoidable process. Traditional procedures for facial rejuvenation have multiple disadvantages. In this case report, we used an innovative technique combining thread lift with small incision rhytidectomy for facial rejuvenation. Management for complication was also reported.

**Patient concerns:**

We presented a 52-year-old male with facial ptosis and wrinkles.

**Diagnoses:**

The patient was diagnosed as facial aging including skin laxity, mid-face and mandibular jowl ptosis, static crows-feet wrinkles, and deepening nasolabial fold.

**Interventions:**

We used an innovative technique combining thread lift with small incision rhytidectomy to treat facial aging.

**Outcomes:**

Improvements of the crow's feet, nasolabial fold, mid-face and lower face ptosis were observed. Complication of subcutaneous nodule was corrected with cosmetic effect of thread lift remained.

**Lessons:**

The innovative technique combining thread lift with small incision rhytidectomy is a good alternative for the treatment of facial aging.

## 1 Introduction

Thread lift, as a minimal invasive facial rejuvenation procedure, has become increasingly popular all over the world.^[1]^ Here we introduced an innovative technique combining absorbable thread lift with small incision rhytidectomy for patients with excess skin. For these patients, thread lift alone could not achieve desired results. Management for complication was also reported.

## 2 Case presentation

**Figure 1:**
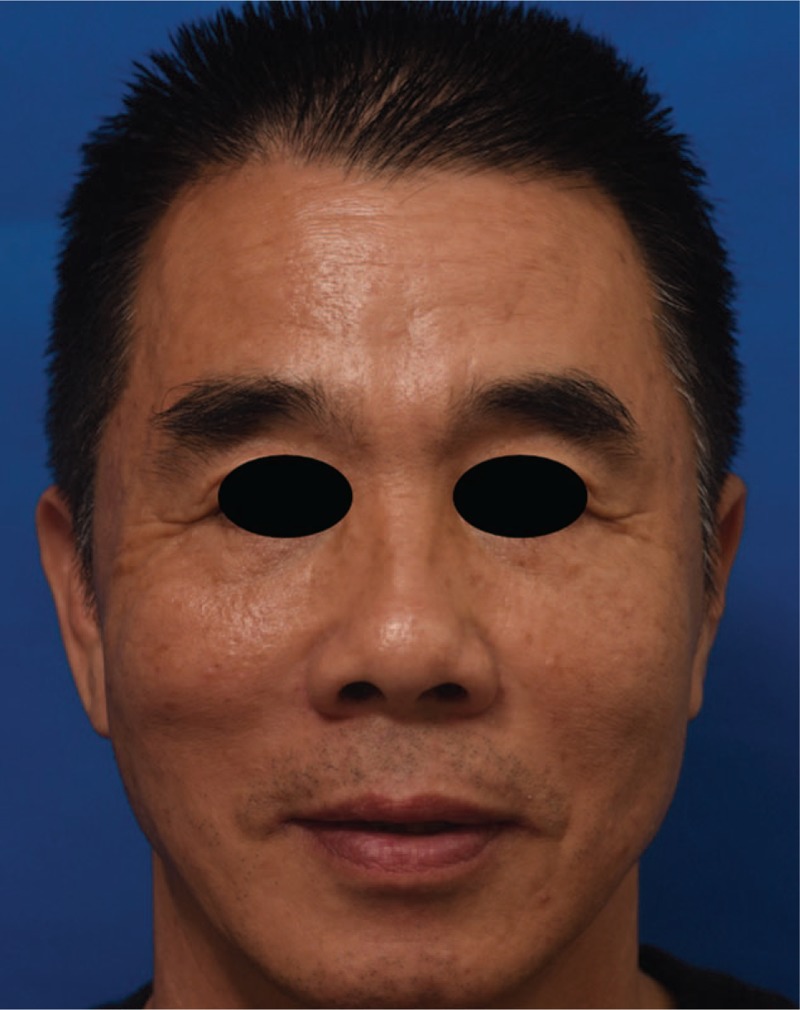
A 52-year-old male presenting with skin laxity, mid-face and mandibular jowl ptosis, static crows-feet wrinkles, and deepening nasolabial fold.

A 52-year-old male presented for a cosmetic consultation. He had skin laxity, mid-face and mandibular jowl ptosis, static crows-feet wrinkles, and deepening nasolabial fold (Fig. 1). The patient was prone to receive procedure with minimal trauma. Considering skin laxity, we recommended this innovative technique combining thread lift with small incision rhytidectomy.

The patient had not received any treatment before. He underwent clinical assessment and routine preoperative examinations. Written informed consent was obtained from the patient. The principles of the 1975 Declaration of Helsinki were followed.

Thread line were designed and marked before the procedure. The surgical procedure was performed under local anesthesia. According to the markings, small needle knife^[2]^ was inserted at superficial muscular aponeurotic system (SMAS) layer to break ligaments and make pilot tunnels for thread cannulas. Absorbable poly(*p*-dioxanone) (PPDO) threads (Tianjin Dongnan Hengsheng Medical Technology, Tianjin, China.) with the following specifications were used: bidirectional barbed, 19 gauge, 150 mm length with blunt needle. Five threads were inserted on each side between the lateral aspect of nasolabial fold and laterally preauricular aspect in an oblique manner. Before removing cannulas, we manually pulled threads and the tissue to the lifted position. Genital pressure was applied over the skin to anchor the barbed thread inside the tissues. A small preauricular incision was made to excise excess skin and closed with 6-0 Prolene suture. The results were assessed objectively with serial photography and subjectively based on the patient's satisfaction.

**Figure 2:**
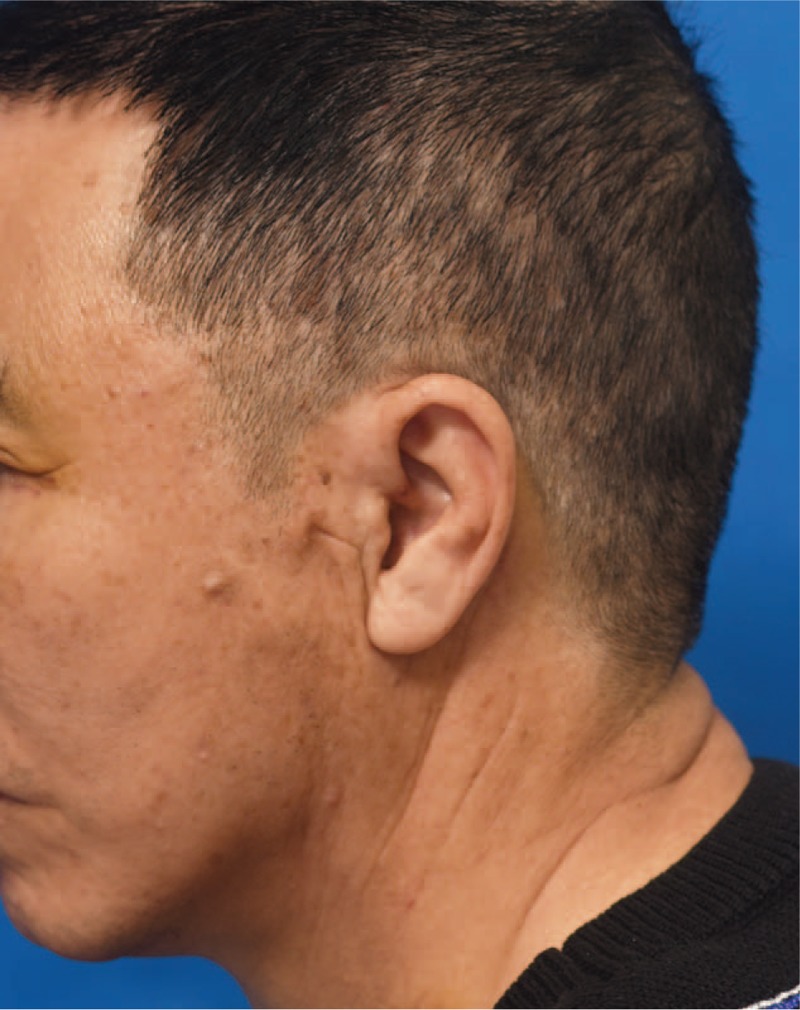
Ten days after the procedure, the patient presented with subcutaneous nodule and palpable knot at the left side of his face.

**Figure 3:**
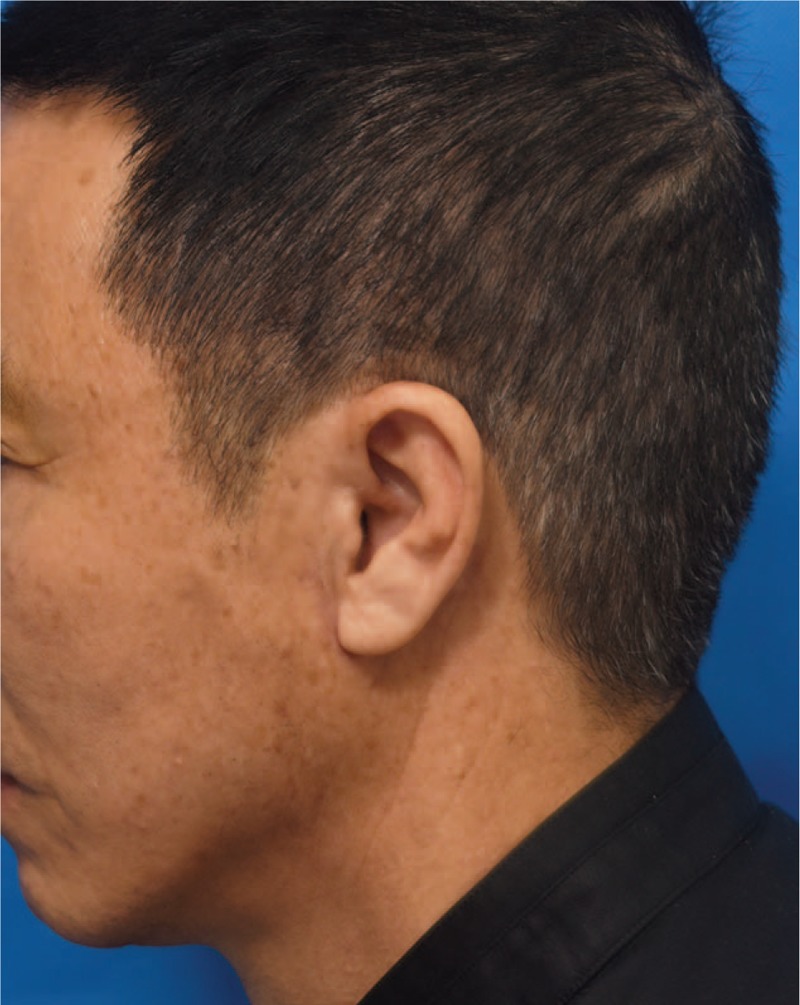
One month after the procedure, the nodule and knot were corrected after small needle knife dissection with almost invisible scar.

Following the procedures, improvements of the crow's feet, nasolabial fold, and mid-face and lower face ptosis were observed. However, 10 days after the procedure, he complained of subcutaneous nodule with palpable knot at the left side (Fig. 2). The patient did not feel pain or other discomfort. After discussing management options, which included observation, open surgical removal and so on, we attempted a minimal invasive approach. Under local anesthesia, small needle knife was inserted to break the fibrosis around the knot without cutting the thread. The fixation knot was preserved and 1 month later, the nodule was flattened and the knot was no longer palpable (Fig. 3).

**Figure 4:**
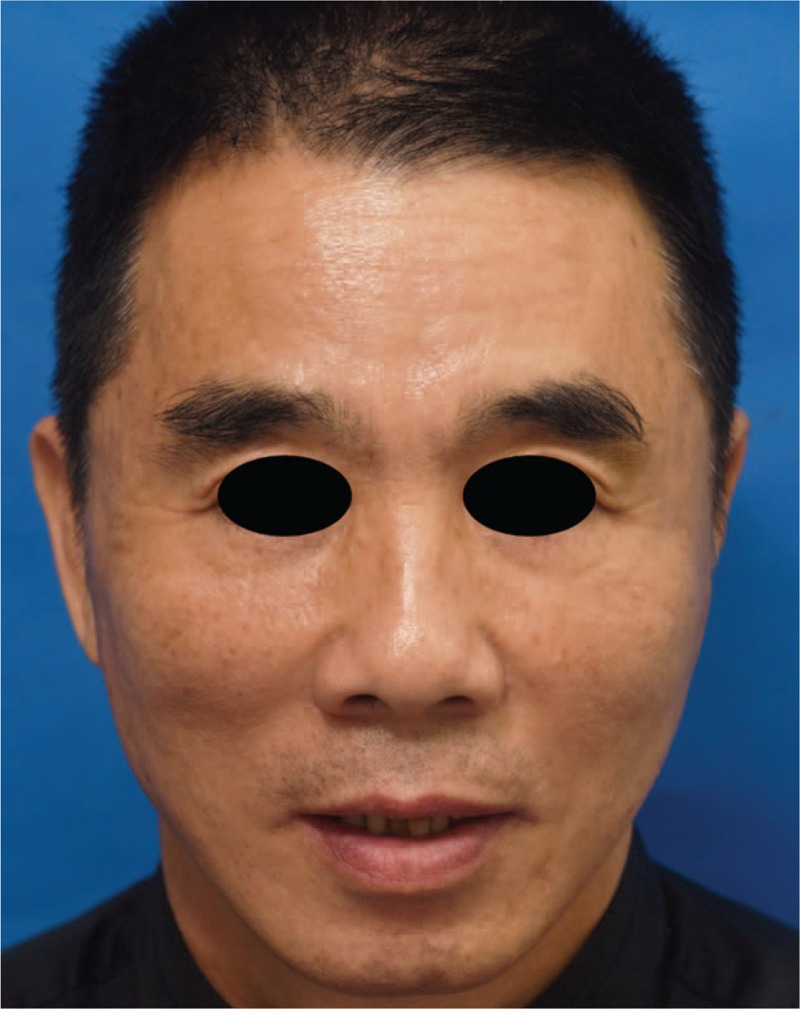
The cosmetic effect of thread lift remained and the skin quality was improved 3 months after the procedure.

The patient tolerated the procedure well without bleeding or other complications. The incisions of rhytidectomy and small needle knife healed with nearly invisible scar. The cosmetic effect of thread lift remained and the skin quality was improved 3 months after the procedure (Fig. 4).

## 3 Discussion

Aging of face is a progressive and inevitable process. Various surgical and nonsurgical procedures have been adopted for facial rejuvenation. For patients with ptosis and facial laxity, surgical face-lift was a traditional method of rejuvenation. Despite its effectiveness, face-lift has multiple disadvantages of obvious scar, great trauma, nerve damaging risks, and long recovery period.^[3]^

As physicians and patients progressively shifted toward less invasive procedure, thread lift gained prominence as an alternative to surgical face-lift.^[3]^ Previously, nonabsorbable poly-polypropylene threads were proved to have long-term efficacy. However, as time goes on, complications appeared as thread migration and exposure, nerve involvement, foreign body reactions, granuloma formation, and the safety in the long run was doubtable.^[1]^ Recently, absorbable threads made of polydioxanone or other degradable materials were available and applied in facial rejuvenation.^[1]^ Histopathological studies indicated collegan deposition which may cause skin quality improvement and fibrotic reaction for long-term lifting effect.^[4,5]^

For patients with mild ptosis and enough skin elasticity, thread lift could achieve ideal rejuvenation and recontouring results.^[5]^ While for those with moderate to severe ptosis and skin laxity, skin may remain saggy or even exaggerate after thread lift with excess skin bunching at the lateral side of the face, which is difficult to recover with decreased skin elasticity. We combined thread lift with small incision rhytidectomy to overcome this problem and achieved aesthetic improvements. Compared with traditional face-lift surgery, our technique is easy to perform and less traumatic with almost invisible scar and rapid recovery time. The efficacy of reversing sagging and slipping skin is better than single thread-lift procedure.

Small needle knife has been used to release fibrosis and adhesion in fat grafting of scar and received good effect.^[2]^ Here it is applied for soft tissue dissection before inserting the threads to achieve a more reliable result.^[1]^ It is also used to break the fibrosis around the knot when nodule complication came up. Compared with other managements as cutting or removing thread, we successfully corrected the complication with effect of face lift preserved and no permanent sequelae.

In conclusion, this innovative technique combining absorbable thread lift with small incision rhytidectomy is safe and effective for facial rejuvenation with appropriate patient selection. Small needle knife could be used to assist thread-lifting and correct nodule complication, leading to a better cosmetic outcome.

## Author contributions

**Conceptualization:** Jufang Zhang.

**Data curation:** Song-Jia Tang.

**Funding acquisition:** Yu-Yan Wang.

**Investigation:** Jufang Zhang, Song-Jia Tang, Zhong-Xin Sun, Xiao-Xin Wu, Yu-Yan Wang.

**Methodology:** Jufang Zhang, Song-Jia Tang, Zhong-Xin Sun, Xiao-Xin Wu, Yu-Yan Wang.

**Project administration:** Jufang Zhang, Yu-Yan Wang.

**Resources:** Jufang Zhang, Song-Jia Tang, Zhong-Xin Sun.

**Supervision:** Jufang Zhang.

**Validation:** Jufang Zhang, Song-Jia Tang.

**Writing – original draft:** Jufang Zhang, Song-Jia Tang, Zhong-Xin Sun, Xiao-Xin Wu, Yu-Yan Wang.

**Writing – review & editing:** Jufang Zhang, Song-Jia Tang, Xiao-Xin Wu.
